# Newly appointed medical faculty members’ self-evaluation of their educational roles at the Catholic University of Korea College of Medicine in 2020 and 2021: a cross-sectional survey-based study

**DOI:** 10.3352/jeehp.2021.18.28

**Published:** 2021-11-05

**Authors:** Sun Kim, A Ra Cho, Chul Woon Chung

**Affiliations:** 1 Department of Medical Education, College of Medicine, The Catholic University of Korea, Seoul, Korea; 2 Department of General Surgery, International St. Mary’s Hospital, Catholic Kwandong University College of Medicine, Incheon, Korea; Hallym University, Korea

**Keywords:** Curriculum, Educational personnel, Medical education, Medical faculty, Republic of Korea

## Abstract

This study aimed to investigate the degree to which newly appointed medical faculty members at the Catholic University of Korea are aware of Harden and Crosby’s 12 educational roles and to identify their preferred educational roles. A 12-item survey questionnaire was distributed to 110 participants, and 100 responses were included in the analysis. The respondents gave the highest score to “clinical or practical teacher” and the lowest score to “curriculum planner” for their current personal competencies. For their preferred personal future competencies, they assigned the highest score to “on the job role model” and the lowest score to “student assessor.” They gave almost equally high values to all 12 roles. However, individual faculty members had different preferences for educational roles. Accordingly, medical schools need to plan and implement customized faculty development programs, and efforts to provide appropriate educational roles according to individual faculty members’ preferences are needed.

## Background/rationale

In the 21st century, medical education is rapidly changing from a teacher-centered to a student-centered framework. Changes in medical education can be largely divided into those affecting the curriculum, teaching methods, and educational evaluation methods. Curricula began to emphasize the importance of outcome-based education in the 2000s [1]. The main changes in teaching methods have included problem-based learning aimed at learners’ problem-solving skills, judgment, and analytical skills [2,3], team-based learning, which emphasizes cooperative learning, and flipped learning, which allows students to learn basic knowledge before class [4,5]. Changes in educational evaluation have included performance evaluations such as objective structured clinical examinations [6-8] and clinical performance examinations [9], which actually evaluate students’ performance in clinical situations, criterion-referenced assessments, which evaluate whether individual students have reached their set outcomes, formative assessments aimed at providing feedback to confirm students’ level of improvement, and progress tests, which check the degree to which students improve as they progress through the program [10,11]. The changes in medical education as described above have led to more demands than ever to change the role of the teacher in medical education [12].

In order to prepare for these changes and for teachers to play their proper roles in students’ education, it is necessary to confirm faculty members’ perceptions about the role of the teacher in medical education. Efforts to improve educational competencies through faculty development programs are important in areas where the teacher’s role is lacking. From the academic institution’s point of view, information on teachers’ perceptions can be used to plan and implement customized faculty development programs to address areas where teachers’ roles in medical education are insufficient.

## Objectives

This study aimed to obtain the results from self-evaluations of newly appointed medical faculty members of the Catholic University of Korea College of Medicine on Harden and Crosby’s 12 roles of a teacher in 2020 and 2021. Specifically, the responses for 12 roles were compared according to 3 points of view: importance to the program, current competencies, and preferred future competencies.

## Ethics statement

This study was approved by the Institutional Review Board (IRB) of Songeui Medical Campus, the Catholic University of Korea (IRB approval no., MC21EIDI0093). A waiver of informed consent was also included in the IRB approval.

## Study design

This is a survey result-based cross-sectional descriptive study.

## Setting

The survey questionnaire was provided in print form after a faculty development workshop (held on February 1, 2021 for participants appointed in 2020 and on May 17, 2021 for participants appointed in 2021).

## Participants

The participants were all 110 newly appointed faculty members of the Catholic University of Korea College of Medicine in 2020 and 2021. Out of 105 questionnaires received, 5 had some missing data and were excluded from the study. The final data analysis was done on 100 questionnaires with adequate data available in the analyzable form. No demographic information was gathered from participants.

## Variables

All 12 items of the measurement tool were analyzed as variables.

## Data source/measurement

The data were participants’ responses to a 12-item survey questionnaire, which consisted of a total of 12 roles classified into 6 categories, each with 2 items, including information provider, role model, facilitator, examiner, planner, and resource developer. This measurement tool was developed by Harden and Crosby [13] on the role of the teacher as given in AMEE Guide No. 20. Permission to use this tool was received from the corresponding author of the tool. The original English form was used for the survey. Since this is a widely used tool, separate validity and reliability testing was not done. The 12 roles were described in the questionnaire and participants were asked to rate, on a 5-point Likert scale, the relevance to the medical school of each of the 12 roles identified where 1=none, 2=little, 3=some, 4=considerable, and 5=great (Table 1).

## Bias

All target subjects were recruited; therefore, there was no bias in selecting participants. The causes of the 5 non-responses and 5 incomplete responses were not sought.

## Study size

All target subjects were recruited for the survey. No sample size estimation was done.

## Statistical methods

The quantitative data collected from this study were analyzed using IBM SPSS ver. 21.0 (IBM Corp., Armonk, NY, USA) to compare the responses according to 3 points of view. The raw data are available from Dataset 1.

## Main results

Newly appointed faculty members assigned the highest score to “clinical or practical teacher” (3.89) and the lowest score to “curriculum planner” (3.08) for their own current personal competencies. They also assigned the highest score to “on the job role model” (4.26) and the lowest score to “student assessor” (3.59) for their own preferred personal future competencies (Table 1). Table 2 presents a comparison between current personal commitment scores and preferred personal future commitment scores. Statistically significant differences were noted in responses for the 12 roles (P<0.05 in all cases).

New faculty members were asked about the relative importance of roles to the medical school teaching program. It is evident from Table 1 that all the scores were similar to each other, varying from 3.91 to 4.40. This indicates that new faculty members gave almost equally high values to all 12 roles.

## Key results

Newly appointed faculty members evaluated their current educational dedication at the “some” level, with an average of 3 points (out of a maximum of 5 points). There was also a difference in the evaluation of the importance of educational roles, with scores ranging from the upper 3 points to the 4 points range. Therefore, it can be seen that among the 12 educational roles, there were differences in preferences for each role.

## Interpretation

The findings of this study indicate that in medical schools, efforts will be needed to assign tasks to match each faculty members’ preferred educational role. To this end, it will first be necessary to identify more specifically each faculty member’s preferred educational role before they begin teaching. Since newly appointed faculty members evaluated their current competencies as insufficient to perform their preferred educational roles, it is necessary to provide customized medical school teaching programs that would reflect each faculty member’s preferences. Second, the preferred future educational competency scores were relatively high compared to the current levels of educational competencies. Therefore, while providing personalized medical school teaching programs, faculty development should be continued with a focus on competencies evaluated as insufficient among the basic 12 roles of a teacher. This means that faculty members in medical schools cannot only play their preferred educational roles; instead, since they must play various educational roles, medical school teaching programs should conduct competency development for areas where they lack competency. Third, the responses regarding the importance of the 12 roles of teachers confirmed that most of the educational roles were perceived to be important, with an average of 4 points. The highest average scores for importance were given for “clinical or practical teacher as an information provider” and “on the job role model” (4.40 and 4.35, respectively). This finding suggests that newly appointed faculty members emphasized the importance of clinical education.

## Limitations

It is difficult to generalize this study’s results, which are based only on a survey of newly appointed faculty members at a single medical school with a relatively small sample size. Therefore, further research at multiple institutions may also be needed for the development of teaching programs based on a better understanding of teachers’ roles.

## Conclusion

It is essential to plan a medical school teaching program that addresses Harden and Crosby’s 12 important educational roles and to provide educational opportunities to faculty members. For educational roles evaluated as more important, corresponding educational content should be included in the stage of planning a medical school teaching program. In addition, medical schools need to continue to improve their medical school teaching programs by grasping to what extent faculty members are developing educational competencies through medical school teaching programs and specifically applying them to the educational field.

## Figures and Tables

**Table 1.** Mean scores of 100 new faculty members for the 12 roles of the medical teacher

Category	12 roles	Current personal competencies^a)^	Importance to medical school teaching program	Preferred personal future competencies
Information provider	Lecture in classroom setting	3.55±0.85	4.05±0.77	3.75±0.93
	Clinical or practical teacher	3.89±0.81	4.40±0.68	4.20±0.79
Role model	On the job role model	3.84±0.81	4.35±0.71	4.26±0.77
	Teaching role model	3.58±0.91	3.97±0.78	3.95±0.90
Facilitator	Mentor, personal advisor	3.58±1.00	4.09±0.72	4.07±0.80
	Learning facilitator	3.43±1.00	4.09±0.74	3.94±0.89
Examiner	Student assessor	3.36±1.00	3.94±0.79	3.59±0.92
	Curriculum evaluator	3.10±1.16	3.91±0.84	3.67±0.94
Planner	Curriculum planner	3.08±1.29	4.02±0.84	3.69±0.98
	Course organizer	3.13±1.25	4.09±0.84	3.71±0.97
Resource developer	Study guide producer	3.18±1.21	4.03±0.87	3.69±0.96
	Resource material creator	3.22±1.20	4.08±0.86	3.76±1.01

Values are presented as mean±standard deviation.

a) In this table, “compentencies” are used instead of “commitments,” as mentioned in the original tool, because the questionnaire items dealt with competencies, not commitments.

**Table 2.** Comparison of the distribution of responses between current personal competencies and preferred personal future competencies

Category	12 roles	Paired differences		t-value	df	Signed 2-tailed
		Mean±SD	95% CI of the difference			
Information provider	Lecture in classroom setting	-0.200±0.778	-0.354 to -0.046	-2.569	99	0.012
	Clinical or practical teacher	0.310±0.647	-0.438 to -0.182	-4.794	99	0.001
Role model	On the job role model	0.420±0.713	-0.562 to -0.278	5.889	99	0.001
	Teaching role model	-0.370±0.837	-0.536 to -0.204	-4.422	99	0.001
Facilitator	Mentor, personal advisor	0.490±0.798	-0.648 to -0.332	-6.143	99	0.001
	Learning facilitator	0.510±0.835	-0.676 to -0.344	-6.109	99	0.001
Examiner	Student assessor	0.230±0.750	-0.379 to -0.081	-3.066	99	0.003
	Curriculum evaluator	0.570±0.967	-0.762 to -0.378	-5.897	99	0.001
Planner	Curriculum planner	0.610±1.024	-0.813 to -0.407	-5.958	99	0.001
	Course organizer	0.580±0.934	-0.765 to -0.395	-6.210	99	0.001
Resource developer	Study guide producer	0.510±0.937	-0.696 to -0.324	-5.441	99	0.001
	Resource material creator	0.540±0.881	-0.715 to -0.365	-6.129	99	0.001

SD, standard deviation; CI, confidence interval; df, degrees of freedom.

## Footnotes

**Data availability**
Data files are available from Harvard Dataverse: https://doi.org/10.7910/DVN/EVYTJP Dataset 1. Raw response data from 100 participating new faculty members of the Catholic University of Korea in 2021.

## Supplementary materials

Data files are available from Harvard Dataverse: https://doi.org/10.7910/DVN/EVYTJP

Supplement 1. Audio recording of the abstract.
